# Integrating Alzheimer's Messages into Chronic Disease Programs

**DOI:** 10.1093/geroni/igab046.2762

**Published:** 2021-12-17

**Authors:** Leslie Best, Rebecca Drewette Card, Lisa Garbarino, Christopher Taylor, Kimberly Boim, Benjamin Olivari

**Affiliations:** 1 National Association of Chronic Disease Directors, Decatur, Georgia, United States; 2 Public Health Partners, Topsham, Maine, United States; 3 Centers for Disease Control and Prevention (CDC), Atanta, Georgia, United States; 4 Centers for Disease Control and Prevention (CDC), Atlanta, Georgia, United States; 5 CDC, Atlanta, Georgia, United States

## Abstract

The number of people in the United States with dementia is increasing, with nearly six million people living with Alzheimer’s disease and related dementias. It is the fifth leading cause of death for those aged ≥65 years. Over 95% of people with dementia have another comorbid chronic condition. The Healthy Brain Initiative’s State and Local Public Health Partnerships to Address Dementia: The 2018-2023 Road Map notes that public health agencies should raise awareness of the link between brain health and physical health, and specifically calls out tobacco prevention and control, cardiovascular health management diabetes prevention and management, obesity prevention and control, and injury prevention as intervention points. The National Association of Chronic Disease Directors developed brain health messages targeted to reduce risk for cognitive decline through the prevention and control of comorbid chronic conditions. These messages can be leveraged for public health action by integrating them into existing chronic disease programs.

